# Best practice advice for asthma exacerbation prevention and management in primary care: an international expert consensus

**DOI:** 10.1038/s41533-024-00399-2

**Published:** 2024-11-17

**Authors:** Neil Skolnik, Barbara P. Yawn, Jaime Correia de Sousa, María Mar Martínez Vázquez, Amanda Barnard, Wendy L. Wright, Austin Ulrich, Tonya Winders, Stephen Brunton

**Affiliations:** 1 Thomas Jefferson University, Philadelphia, PA USA; 2 Jefferson Health, Philadelphia, PA USA; 3 University of Minnesota, Minneapolis, MN USA; 4 University of Minho, Braga, Portugal; 5 University of the Basque Country, Leioa, Spain; 6 International Primary Care Respiratory Group (IPCRG), Scotland, UK; 7 Australian National University, Canberra, ACT Australia; 8 Wright & Associates Family Healthcare, Amherst, MA USA; 9 Partners in Healthcare Education, PLLC, Amherst, MA USA; 10 Primary Care Education Consortium, Winnsboro, SC USA; 11 Global Allergy & Airways Patient Platform, Vienna, Austria; 12 US Primary Care Respiratory Group, Winnsboro, SC USA

**Keywords:** Asthma, Therapeutics

## Abstract

Primary care clinicians play a key role in asthma and asthma exacerbation management worldwide because most patients with asthma are treated in primary care settings. The high burden of asthma exacerbations persists and important practice gaps remain, despite continual advances in asthma care. Lack of primary care-specific guidance, uncontrolled asthma, incomplete assessment of exacerbation and asthma control history, and reliance on systemic corticosteroids or short-acting beta_2_-agonist-only therapy are challenges clinicians face today with asthma care. Evidence supports the use of inhaled corticosteroids (ICS) + fast-acting bronchodilator treatments when used as needed in response to symptoms to improve asthma control and reduce rates of exacerbations, and the symptoms that occur leading up to an asthma exacerbation provide a window of opportunity to intervene with ICS. Incorporating patient perspectives and preferences when designing asthma regimens will help patients be more engaged in their therapy and may contribute to improved adherence and outcomes. This expert consensus contains 10 Best Practice Advice Points from a panel of primary care clinicians and a patient representative, formed in collaboration with the International Primary Care Respiratory Group (IPCRG), a clinically led charitable organization that works locally and globally in primary care to improve respiratory health. The panel met virtually and developed a series of best practice statements, which were drafted and subsequently voted on to obtain consensus. Primary care clinicians globally are encouraged to review and adapt these best practice advice points on preventing and managing asthma exacerbations to their local practice patterns to enhance asthma care within their practice.

## Introduction

**Fig. 1:**
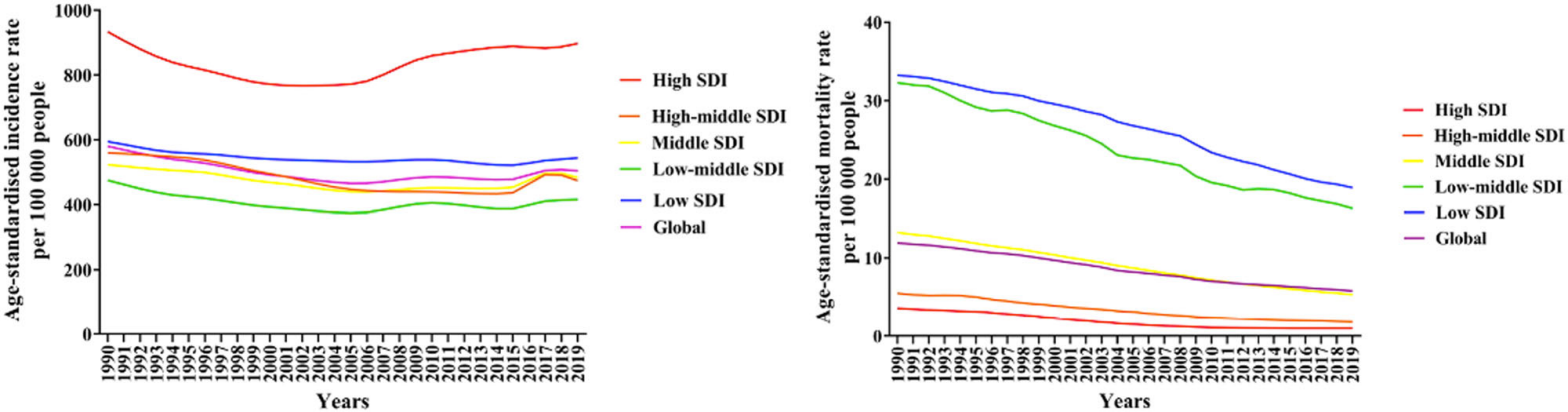
Global age-adjusted incidence and mortality rates of asthma by sociodemographic index^4^. SDI, sociodemographic index. Reproduced without modification from: Cao Y, et al. *Front Public Health*. 2022;10:1036674 under Creative Commons Attribution 4.0 International License (https://creativecommons.org/licenses/by/4.0/legalcode).

Asthma exacerbations (also referred to as “flare-ups” or “attacks”) are characterized by a sudden or progressive worsening of symptoms including wheezing, chest tightness, shortness of breath, cough, and decline in lung function. These are an alteration from the patient’s usual condition, necessitating a change in therapy^1^. Asthma exacerbations are a significant cause of disease-related morbidity, and mortality, progressive loss of lung function, and increased health care costs globally^2^. Some data over the past decades show reductions in age-adjusted asthma mortality, while asthma incidence remains steady or slightly increased (Fig. 1). Asthma exacerbation rate data are inconsistent, with some showing increased exacerbations and some showing decreased exacerbations in recent years^3,4^, but emergency department (ED) visits for asthma remain a significant challenge^5,6^. Further, asthma exacerbations may be underreported as they are not always medically treated or reported^7^.

**Fig. 2:**
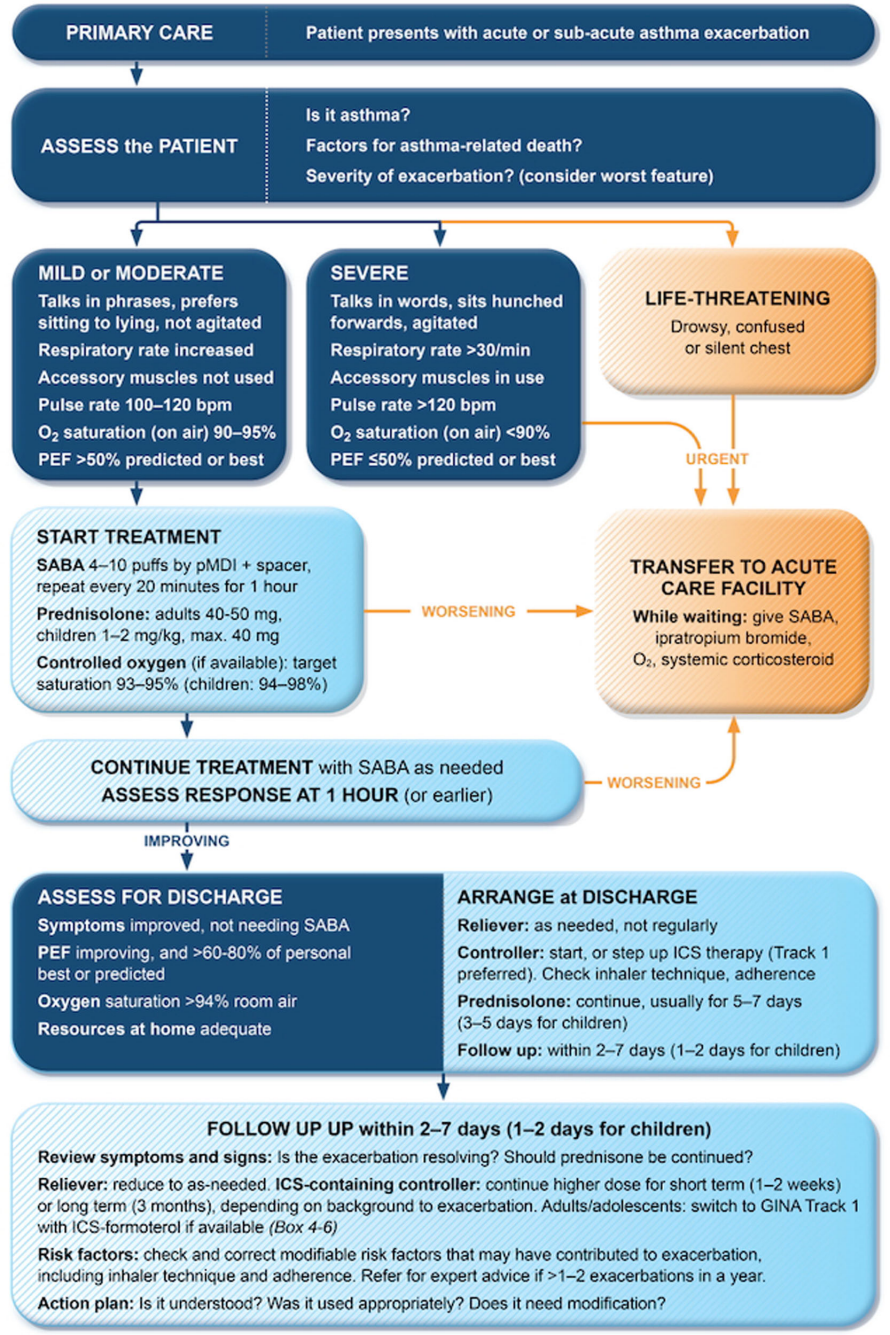
Management of asthma exacerbations in primary care (GINA)^1^. Source: From GINA ©2024 Global Initiative for Asthma, reprinted with permission. Available from www.ginasthma.org.

Primary care clinicians (PCCs) play an integral role in preventing and managing asthma exacerbations and most patients can be successfully managed in primary care worldwide^1,8–10^. Enhancing prevention and management of asthma exacerbations in primary care settings would be expected to further lower asthma morbidity and potentially mortality. The Global Initiative for Asthma (GINA) report includes an algorithm for exacerbation recognition and management in primary care, which can facilitate implementation of practical step-by-step strategies to deal with exacerbations (Fig. 2)^1^.

This international expert consensus seeks to develop practice-specific recommendations for asthma exacerbation diagnosis and management for primary care by building on available resources including GINA, Canadian Thoracic Society recommendations, the National Asthma Education and Prevention Program (NAEPP) in the United States, guidance from the Japanese Society of Allergology, and the Australian Asthma Handbook, among others^1,10–14^. The consensus best practice statements are listed in the Table 1 and further explored below.

**Table. 1:** Best practice advice consensus statements from the international expert panel.

**Identification and Assessment of Asthma Exacerbations**
• BPA 1: Consider incorporating available validated tools into primary care settings to evaluate asthma status including symptom burden, exacerbation history, and risk. • BPA 2: Counsel patients on the warning signs and symptoms of loss of asthma control that may precede exacerbations to facilitate initiation of timely and effective treatment to prevent exacerbations or reduce their severity. • BPA 3: Recognize and support education and management plans addressing exacerbation risk for people with all severities of asthma. • BPA 4: Evaluate adherence to prescribed therapy (target adherence ≥75%) and inhaler technique at all asthma related visits, asking non-judgmental questions, and provide education and support based on that evaluation.
**Asthma Exacerbation Management and Prevention**
• BPA 5: Recognize the cumulative adverse effects of systemic corticosteroids (SCS) use and work to avoid their overuse by preventing future exacerbations. • BPA 6: Consider the use of AIR, MART (formerly known as SMART), or ICS-SABA quick reliever regimens for treating asthma and exacerbations to address underlying inflammation as well as provide bronchodilation. • BPA 7: After an exacerbation, request a patient follow up visit within a short time to explore steps to prevent future exacerbations; these may include providing self-management education, inhaler technique review, adherence evaluation, smoking cessation advice, an updated asthma action plan, and updating immunizations.
**Access to Asthma Care and Treatments**
• BPA 8: Seek to improve timely access to asthma care and treatments to reduce delays in exacerbation prevention and management. • BPA 9: Seek to incorporate patients’ and families’ perspectives, preferences, and goals into asthma care. • BPA 10: Encourage and participate in multidisciplinary team-based care of patients with asthma to ensure continuity of care and improved outcomes.

*BPA* best practice advice.

## Methods

A multi-nation expert panel of individuals with expertise and experience in asthma management in primary care practice was assembled. The panel included clinicians from the United States, Australia, Spain, and Portugal, and a patient advocate. The panel was formed in collaboration with the International Primary Care Respiratory Group (IPCRG), a clinically led charitable organization that works locally and globally in primary care to improve respiratory health. IPCRG’s 155,000+ members are based in 40 different countries. The panel met virtually and developed a series of best practice statements, which were drafted and subsequently voted on to obtain consensus. To reach consensus, the expert panel members participated in a survey, requiring a predefined threshold of 75% approval for each Best Practice Advice point. An initial failure to reach consensus was resolved by subsequent discussions, revisions as needed, and re-voting.

## Identify and Assess Asthma Exacerbations

### Best Practice Advice 1: Consider incorporating available validated tools into primary care settings to evaluate asthma status including symptom burden, exacerbation history, and risk

Assessing asthma control is fundamental for asthma management to optimize medication therapy, prevent exacerbations, improve quality of life and achieve patient and clinical treatment goals^1,15^. Clinicians’ and patients’ assessment of asthma control tends to overestimate control and often differ from each other. Validated tools can help improve the accuracy of assessment of asthma control^16^. However, most validated tools assess only symptoms (shortness of breath, wheezing, coughing, chest tightness or pain) with little or no attention to past exacerbations and therefore the risk of exacerbations (also called “asthma attacks”) which is also a key factor in a patient’s overall asthma control^17^.

An ideal tool for assessing asthma control should include questions that reveal both symptoms and exacerbation risk, such as the Asthma Impairment and Risk Questionnaire (AIRQ)^18,19^. Prior exacerbations are the best predictor of future exacerbations, which is one reason the AIRQ contains questions focusing on exacerbation history.

Validated asthma assessment tools include the following:

- AIRQ. The AIRQ is a recently developed and validated 10 “yes/no” question tool that incorporates both symptom and exacerbation risk assessment^17,18^. Scores range from 0–10, with a score of 0–1 indicating well-controlled asthma and higher scores representing worsening asthma control^18^. AIRQ control level has been found to predict risk of future exacerbations over the following 12 months^19^. The assessment tool is linked to suggestions for further evaluation of each question domain. Between annual visits, a follow-up version of AIRQ can be used to assess ongoing disease status and the impact of interventions^20^.
  ○ Link to AIRQ: https://www.asthmaresourcecenter.com/home/for-your-practice.html
- Asthma APGAR. The Asthma APGAR includes 6 questions with 2-week recall; the 3 multi-answer questions address symptoms and activity limitations and are scored with the other 3 to identify potential reasons for lack of control. Scores of >2 are considered inadequate control. It is linked to a care algorithm based on NAEPP guidelines^21,22^.
  ○ Link to Asthma APGAR questions and care algorithm: https://www.aafp.org/dam/AAFP/documents/patient_care/nrn/nrn19-asthma-apgar.pdf.
- Asthma Control Test (ACT). The ACT includes 5 multi-answer questions about symptoms, activity limitations, rescue inhaler use and patient perception of control with 4-week recall. Scores range from 5–25 with higher scores indicating better control^23^. A score of 20–25 indicates well-controlled asthma, and the maximum clinically important difference is 3 points^24^.
  ○ Link to ACT questions: https://www.asthmacontroltest.com/welcome
- Asthma Control Questionnaire (ACQ). The ACQ includes 5 symptom-based questions with 4-week recall^1,25^. Scores range from 0–6, with higher scores indicating worse asthma control; the total score is an average of individual items^1^.
  ○ Link to obtain ACQ: https://www.qoltech.co.uk/acq.html
- Control of Allergic Rhinitis and Asthma Test (CARAT). CARAT is a 10-question patient‐reported outcome measurement (PROM) assessing the control of asthma and allergic rhinitis at a 4-week interval. Scores range from 0 to 30. Scores higher than 24 indicate good disease control^26^. There are separate scores for asthma and allergic rhinitis.
  ○ Link to obtain CARAT: https://www.new.caratnetwork.org/fastcarat/index.html

The GINA report includes a suggestion for 4 areas to be covered when assessing control. The questions are not validated but are a good guide to what to ask if a validated questionnaire is not used.

- Link to GINA questions: (page 15) https://ginasthma.org/wp-content/uploads/2020/04/Main-pocket-guide_2020_04_03-final-wms.pdf

Using validated tools in practice requires planning to implement but has been reported to save clinician time in continuity of care^22^. Practical implementation strategies might include asking patients to complete questions before seeing the clinician, with assistance from the receptionist, rooming staff, or an online portal. The clinician will then be able to quickly review the results and incorporate them into treatment decisions, without using time during the appointment to conduct the assessment. The validated tools and GINA questions can ensure the needed information is obtained, compared to asking less useful questions such as “How is your asthma?” Scores can be followed over time to assess treatment effectiveness. Mobile health apps may also be useful to facilitate asthma self-management and symptom awareness^27^.

### Best Practice Advice 2: Counsel patients on the warning signs and symptoms of loss of asthma control that may precede exacerbations to facilitate initiation of timely and effective treatment to prevent exacerbations or reduce their severity

Counseling patient on warning signs such as an increase in their usual asthma symptoms or new onset of things such as cough that can precede exacerbations could prompt treatment with anti-inflammatory therapy, help mitigate exacerbation severity, and potentially prevent an exacerbation from occurring^2,28^. Appropriate use of anti-inflammatory therapy (ICS) prior to an exacerbation may decrease use and overuse of health care resources such as the ED or urgent care, SABA and systemic corticosteroids (SCS).

Using an asthma action plan can give patients and families specific parameters for patients to take action in identifying and using early treatment for an exacerbation^29^. Exacerbation triggers and how to address them should be identified.

While spirometry is the gold standard for diagnosing asthma it has limited practical value in exacerbation management^1^. Peak expiratory flow (PEF) measurement can provide objective data on lung function for assessing exacerbation severity and response to treatment^30^. However, frequency and severity of symptoms is a more practical and widely available measure of exacerbation onset and is more sensitive than PEF for most people^31^. For those with poor perception of airflow limitation symptoms, regular PEF monitoring can help proactively identify exacerbation episodes^1,32^.

#### Window of opportunity for intervention

About 10–14 days before an asthma exacerbation, progressively rising inflammation often underlies the decrease in lung function (PEF), accompanied by an increase in symptoms^33,34^, which may result in patients increasing SABA use^34–36^. SABA use can provide symptomatic relief, but it does not address airway inflammation and overuse of SABA has been shown to increase risks^33,34^. The timeframe leading up to an exacerbation may represent a “window of opportunity” to minimize airway inflammation and either prevent or reduce the exacerbation by adding anti-inflammatory therapy, if the patient is not using anti-inflammatory therapy, or scaling up the current anti-inflammatory dose.

GINA recommends the use of an anti-inflammatory reliever (AIR), which is low dose as-needed ICS-formoterol, or ICS-SABA for symptom control rather than SABA-only as a means to improve control and mitigate the risk of a serious exacerbation. Formoterol has the advantage of a fast and long-acting bronchodilator, while salbutamol (albuterol) is also fast but short-acting^1^.

### Best Practice Advice 3: Recognize and support education and management plans addressing exacerbation risk for people with all severities of asthma

Asthma exacerbations can occur in all severities of asthma despite guideline-directed treatment^2^. A history of ED visits or hospitalization for an exacerbation increase the risk of future exacerbations, irrespective of severity, patient demographics, or clinical characteristics^2,37^. Patients with intermittent, mild, and moderate asthma are all at risk for exacerbations, which is often related to unrecognized lack of asthma control.

Recently in the US, approximately 60% of adults and 44% of children were reported to have uncontrolled asthma^38,39^, with more than 80% of whom had mild or moderate asthma^40^. In an international cohort of 1115 patients classified as GINA Step 1 or Step 2, 25% had uncontrolled asthma and about 33% reported rescue inhaler use in the previous 4 weeks^1,41^. Based on United Kingdom data from the National Review of Asthma Deaths, up to 45% of patients across asthma severities dies without seeking medical assistance or before emergency care could be provided, indicating a need for improved education and management plans^42^.

Appropriate and optimal therapy to minimize symptoms, exacerbations risk, and routine exacerbation assessment and history is important for all patients with asthma, regardless of severity.

### Best Practice Advice 4: Evaluate adherence to prescribed therapy (target adherence ≥ 75%) and inhaler technique at all asthma related visits, asking non-judgmental questions, and provide education and support based on that evaluation

Although ICS are highly effective anti-inflammatory therapies for asthma, patients often demonstrate poor adherence to prescribed ICS-containing daily maintenance regimens^43,44^. Those with uncontrolled asthma and inadequate adherence are at the highest risk for adverse outcomes^45^. Adherence rates of ≥75% have been shown to significantly improve asthma control^46^. Assessing adherence can be accomplished using open ended non-judgmental questions such as “It is often hard to take an inhaler every day. How many times a week do you think you miss or forget or cannot take your asthma inhalers?”

Use of SABA-only inhalers for rescue or quick symptom relief can lead to overuse of SABA. The use of an ICS/fast-acting bronchodilator (SABA or fast-acting LABA [long-acting beta agonist]) has been shown to decrease exacerbations compared to the use of albuterol alone^1,47–50^.

Up to 80% of patients with asthma have incorrect inhaler technique, which may be associated with factors such as age, sex, education level and failure of patients to be shown proper technique^51,52^. Incorrect inhaler use has been linked to poor asthma outcomes such as more frequent ED visits and hospitalizations, prescriptions of SCS and antibiotics (overused in asthma exacerbation management), and worsened disease control^51,53^. Even after successful intervention to improve inhaler technique, patients may revert to incorrect use within a short time requiring repeated teaching and evaluation updates^51,54^.

Given the importance of correct inhaler technique for asthma control and exacerbation prevention, all healthcare professionals such as physicians, nurses, and pharmacists should be involved in the instruction and review of inhaler technique. Repeated assessment at each visit and education on correct methods is recommended and may yield benefits for individual patients without risk of harm^1,51^. Several resources are available to help teach inhaler technique.

- IPCRG inhaler videos: https://www.ipcrg.org/resources/inhaler-resources
- Australia National Asthma Council videos: https://www.nationalasthma.org.au/living-with-asthma/how-to-videos

## Actively Address Asthma Exacerbation Management and Prevention

### Best Practice Advice 5: Recognize the cumulative adverse effects of systemic corticosteroids (SCS) use and work to avoid their overuse by preventing future exacerbations

While some asthma exacerbations may require SCS^1,10^, earlier recognition and use of ICS with SABA or fast-acting LABA for quick relief or early in an exacerbation may mitigate the need for SCS. Rarely, if ever, are SCS required for effective maintenance management, and their use should be minimized where possible due to potential short-term and long-term adverse effects^55^.

Adverse effects resulting from SCS use occur based on cumulative lifetime dose, starting at doses as low as 500 mg of prednisone or equivalent and less than 30 days of exposure^56^. Adverse effects of SCS can occur with both chronic and repeated episodic use with cumulative doses ≥1000 mg of prednisone equivalent per year, regardless of the length of treatment. A common regimen for exacerbation management is prednisone (or equivalent) 40–60 mg for 5–10 days, adding up to a cumulative dose of 200–600 mg per exacerbation, approaching or exceeding the long-term effect risk threshold after even a single course of SCS^1,10^. Higher cumulative SCS doses are associated with increases in cardiovascular disease, osteoporosis, fractures, cerebrovascular disease, pneumonia, kidney impairment, cataracts, sleep apnea, depression, anxiety, type 2 diabetes, and weight gain^56–59^.

To limit the use of SCS, PCCs can implement asthma treatment that appropriately uses ICS-based therapies in both acute and maintenance regimens, thereby reducing the risk of exacerbations and the need for SCS^1,47^. PCCs should include monitoring patients for adverse effects of corticosteroids, especially among those with multiple courses of SCS therapy over the years of their asthma care.

### Best Practice Advice 6: Consider the use of AIR, MART (formerly known as SMART) or ICS-SABA quick reliever regimens for treating asthma and exacerbations to address underlying inflammation as well as provide bronchodilation

For patients requiring maintenance treatment (GINA Steps 3–5), maintenance-and-reliever therapy (MART), formerly known as single-inhaler maintenance-and-reliever therapy (SMART), is referred to by the GINA report as a “treatment regimen in which the patient uses an ICS-formoterol inhaler every day (maintenance dose), and also uses the same medication as needed for relief of asthma symptoms (reliever doses).”^1^ The clinical rationale for recommending MART, or a combination of ICS and fast-acting bronchodilator, is based on the increased risk of severe or fatal exacerbations with SABA-only use, as well as evidence showing a lower frequency of exacerbations with ICS + formoterol as maintenance and rescue therapy^1,60–66^.

For patients only requiring reliever treatment (Steps 1–2), GINA recommends the use of AIR, or low dose as-needed ICS-formoterol, as the preferred treatment (Track 1)^1^.

Use of ICS along with a SABA for rescue therapy can reduce exacerbations compared to SABA-only rescue therapy. In the PREPARE trial, adults with moderate-to-severe asthma who were instructed to take ICS every time they used rescue therapy had a lower annualized rate of severe exacerbations than those who weren’t instructed to take ICS with rescue therapy^49^. In the MANDALA randomized, double-blind trial, adults and adolescents with uncontrolled moderate-to-severe asthma receiving albuterol-budesonide as rescue therapy had a significantly lower risk of severe asthma exacerbations than those receiving albuterol alone^47^. Of note, albuterol-budesonide is approved for the as-needed treatment or prevention of bronchoconstriction and to reduce the risk of exacerbations in patients with asthma 18 years of age and older in the United States.

The physiologic rationale for ICS use with bronchodilation to manage exacerbations is related to the more rapid nongenomic effects of ICS, which may not be widely known among clinicians. Historically clinicians were told that ICS time to onset of anti-inflammatory effects took days to occur. Recent evidence indicates a more rapid onset (minutes) of action of ICS due to the complementary mechanisms of nongenomic *and* genomic effects^67,68^.

Implementing AIR and MART for asthma management and exacerbations may be limited by national and local restrictions and product availability. PCCs are encouraged to increase awareness of available options for AIR and MART in their local areas, including the use of SABA-ICS as a combination inhaler. In areas where such products are unavailable, patients may be instructed to take a dose of ICS each time they use a SABA inhaler (ICS and SABA in separate inhalers), though this can be more cumbersome for patients^1^.

Additionally, some patients may receive their current asthma treatment primarily via nebulizer, which does not easily lend to AIR or MART but can be accommodated by ICS-SABA quick relief therapy. In general, nebulizers are not considered the best practice to deliver asthma treatment and should be discouraged. ICS/SABA rescue therapy may be added to any asthma maintenance regimen and newer combination inhaler therapies may facilitate that choice.

### Best Practice Advice 7: After an exacerbation, request a patient follow up visit within a short time to explore steps to prevent future exacerbations; these may include providing self-management education, inhaler technique review, adherence evaluation, smoking cessation advice, and an updated asthma action plan, and updating immunizations

Effective asthma self-management education includes helping patients understand self-monitoring of symptoms and/or lung function (PEF) and their written asthma action plan^1^. A follow up visit after an exacerbation is essential to review any persistent symptoms, assess current therapy, evaluate and manage modifiable risk factors (such as causative triggers like viral infections—especially those preventable by vaccines [e.g., influenza, respiratory syncytial virus [RSV], SARS-CoV-2]—and allergies, continued smoking or smoke exposure, obesity, poor adherence, and poor inhaler technique), recommend indicated immunizations, and update the asthma action plan^1^. Immediately after an asthma exacerbation can be an effective time to reinforce these concepts with patients as they may be more motivated to prevent future exacerbations with the current exacerbation fresh in their mind.

Clinicians treating a patient with an asthma exacerbation in an ER should add anti-inflammatory treatment with ICS to inhaled bronchodilators at discharge and recommend an appointment with the patient’s PCC within a short time (3–4 days) while providing a discharge letter including a written asthma action plan.

Principles of self-management of exacerbations via a written asthma action plan include^1^:

- How to assess symptoms and detect worsening symptoms that may precede an exacerbation early on
- How to assess lung function using PEF (if applicable)
- When and how to increase reliever (ICS plus rapid acting bronchodilator) treatment
- When and how to increase controller therapy
- How to review response to treatment and assess next steps
- When to contact clinician or emergency services

An example asthma action plan can be found on IPCRG’s website at: https://www.ipcrg.org/sites/ipcrg/files/content/attachments/2021-07-14/asthma-action-plan-adult-2021.pdf.

## Access to Asthma Care and Treatments

### Best Practice Advice 8: Seek to improve timely access to asthma care and treatments to reduce delays in exacerbation prevention and management

Access to adequate asthma care and optimal treatments represents a substantial challenge for many patients across the globe, especially in communities and countries with limited resources, leading to avoidable harm^69^. The burden of asthma can uniquely affect patients and families across different age, socioeconomic, and racial and ethnic groups. For example, disparate patient groups may face barriers accessing the health care system in certain countries due to language barriers, cultural barriers, geo-political barriers, lack of familiarity with the health care systems and resources, poverty, and low numbers of PCCs and health systems^70^.

Obtaining the most appropriate asthma medications can be challenging for patients due to cost, cumbersome prescription requirements, and other factors. For example, patients may be prescribed oral corticosteroids to treat an exacerbation since ICS are often more expensive. In other cases, certain treatments are not available due to government, insurer or regulatory restrictions or product supply issues.

Coordinated efforts and advocacy between clinicians, local authorities, and global organizations can complement local resources to improve access to asthma care and treatments for patients with barriers, helping to address inequity. Telemedicine has been increasingly used to care for patients with asthma since the advent of COVID-19, and it can be a valuable adjunct to face-to-face visits, increasing access and increase frequent patient-clinician contact where needed^71^.

### Best Practice Advice 9: Seek to incorporate patients’ and families’ perspectives, preferences, and goals into asthma care

International asthma guidance documents emphasize patient-clinician collaboration for optimal asthma care^1,10^. As clinicians seek to incorporate patients’ and families’ preferences, goals, and perspectives, patients are more likely to be engaged and understand education in self-management potentially leading to reduced asthma morbidity^72,73^. Additionally, shared decision-making in asthma management is associated with improved adherence and asthma outcomes^74^.

Inhalers that combine ICS with bronchodilators that are used as needed can be an effective treatment for some patients with asthma, whereas SABA-only use has been associated with increased exacerbation risk^75^. In the INSPIRE study, adults with asthma taking an ICS or ICS + bronchodilator maintenance therapy desired treatments that work quickly, most used a SABA daily though they were prescribed maintenance treatment, and many thought they did not need daily medication for asthma when they were feeling well^76^.

Nonadherence or preference for only treatments that provide immediate relief can limit treatment effectiveness. Nonadherence is often not just refusal to take medication and clinicians can see this as an opportunity for education, or even adaptation to improve adherence. Lack of adherence may be due to time, costs, fears, or cultural issues.

### Best Practice Advice 10: Encourage and participate in multidisciplinary team-based care of patients with asthma to ensure continuity of care and improved outcomes

Multidisciplinary care in chronic airway diseases such as asthma can improve outcomes for some patients, especially those with more complex or severe disease^77^. Key members of the multidisciplinary team may involve the PCC, specialist and consultant clinicians, nurses, pharmacists, respiratory therapists, and mental health professionals, as well as support staff in the clinic that interact with patients^77^. The entire clinic and each member of the multidisciplinary team should collaborate and have access to the patient’s medical records where possible to ensure continuity of care.

PCCs should consider referring patients with asthma to specialists or consultants when needed, including for the following common reasons^8^:

- Suspected alternative pulmonary diagnosis
- Unable to confirm asthma diagnosis by usual means
- Suspicion of occupational asthma
- Persistently uncontrolled disease
- Severe disease requiring specialized therapy
- Feeling uncomfortable adequately treating a particular patient

Of note, clinicians should recognize that not all episodes of coughing, wheezing, shortness of breath, and other airway symptoms indicate an asthma exacerbation^1^. Furthermore, a diagnosis of asthma should not always be assumed, especially in patients without an initial thorough workup and assessment for asthma. Distinguishing asthma exacerbations from other problems such as laryngeal disorders, vocal cord dysfunction, and dysfunctional breathing can be challenging^1^. Clinicians should consider whether respiratory symptoms truly indicate worsening of underlying asthma or other symptomatology that does not require treatment intensification.

## Conclusion

This international expert consensus identified best practice statements that are intended to facilitate improved prevention and management of asthma exacerbations worldwide. Increased awareness of exacerbation risk, recognizing the risks of SCS and emphasizing the importance of reliever use of ICS as part of exacerbation prevention and management, encouraging patient adherence, and assessing and teaching correct inhaler technique are major themes the expert panel recommends for PCCs to consider implementing.

## Supplementary information

Supplementary Tables

## Data Availability

No datasets were generated or analysed during the current study.

## References

[1] Global Initiative for Asthma. Global Strategy for Asthma Management and Prevention. Available from: www.ginasthma.org (2024).

[2] Castillo, J. R., Peters, S. P. & Busse, W. W. Asthma exacerbations: pathogenesis, prevention, and treatment. *J. Allergy Clin. Immunology: Pract.* **5**, 918–927 (2017). 28689842 10.1016/j.jaip.2017.05.001 PMC5950727

[3] Skolnik, N. Use of ICS and fast-acting bronchodilators in asthma: past, present, and future. *J. Fam. Pract.* **72**10.12788/jfp.0625 (2023). 10.12788/jfp.0625 37549419

[4] Cao, Y. et al. Global trends in the incidence and mortality of asthma from 1990 to 2019: an age-period-cohort analysis using the global burden of disease study 2019. *Front Public Health* **10**, 1036674 (2022). 36483262 10.3389/fpubh.2022.1036674 PMC9723391

[5] Most Recent National Asthma Data | CDC. Accessed March 31, 2023. https://www.cdc.gov/asthma/most_recent_national_asthma_data.htm. January 9, 2023

[6] Mannino, D. M. et al. Surveillance for Asthma—United States, 1980-1999. *Morbidity Mortal. Wkly. Rep.* **51**, 1–13 (2002). 12420904

[7] Suruki, R. Y., Daugherty, J. B., Boudiaf, N. & Albers, F. C. The frequency of asthma exacerbations and healthcare utilization in patients with asthma from the UK and USA. *BMC Pulm. Med.* **17**, 74 (2017). 28449686 10.1186/s12890-017-0409-3 PMC5406966

[8] Wu, T. D., Brigham, E. P. & McCormack, M. C. Asthma in the primary care setting. *Med. Clin. North Am.* **103**, 435–452 (2019). 30955512 10.1016/j.mcna.2018.12.004 PMC6776421

[9] Fletcher, M. J. et al. Improving primary care management of asthma: do we know what really works? *NPJ Prim. Care Respir. Med.* **30**, 29 (2020). 32555169 10.1038/s41533-020-0184-0 PMC7300034

[10] Cloutier, M. M. et al. 2020 focused updates to the asthma management guidelines: a report from the National Asthma Education and prevention program coordinating committee expert panel working group. *J. Allergy Clin. Immunol.* **146**, 1217–1270 (2020). Expert Panel Working Group of the National Heart, Lung, and Blood Institute (NHLBI) administered and coordinated National Asthma Education and Prevention Program Coordinating Committee (NAEPPCC). 33280709 10.1016/j.jaci.2020.10.003 PMC7924476

[11] Yang, C. L. et al. Canadian Thoracic Society 2021 Guideline update: diagnosis and management of asthma in preschoolers, children and adults. *Can. J. Respir. Crit. Care Sleep. Med.* **5**, 348–361 (2021).

[12] Nakamura, Y. et al. Japanese guidelines for adult asthma 2020. *Allergol. Int.* **69**, 519–548 (2020). 32893125 10.1016/j.alit.2020.08.001

[13] Department of Veterans Affairs, Department of Defense. VA/DoD clinical practice guideline for the primary care management of asthma. Published online September 2019. Accessed January 30. https://www.healthquality.va.gov/guidelines/CD/asthma/VADoDAsthmaCPGFinal121019.pdf (2024).

[14] National Asthma Council Australia. Australian Asthma Handbook. Accessed January 31. https://www.asthmahandbook.org.au/ (2024).

[15] EPR⎯3. “Expert Panel Report 3: Guidelines for the Diagnosis and Management of Asthma (EPR⎯2 1997)”. NIH Publication No. 97-4051. Bethesda, MD: U.S. Department of Health and Human Services; National Institutes of Health; National Heart, Lung, and Blood Institute; National Asthma Education and Prevention Program, 2007. Accessed May 12. https://www.nhlbi.nih.gov/sites/default/files/media/docs/EPR-3_Asthma_Full_Report_2007.pdf (2023).

[16] Greenblatt, M., Galpin, J. S., Hill, C., Feldman, C. & Green, R. J. Comparison of doctor and patient assessments of asthma control. *Respir. Med.* **104**, 356–361 (2010). 19900797 10.1016/j.rmed.2009.10.010

[17] Lugogo, N., Skolnik, N., Jiang, Y. A Paradigm Shift for Asthma Care. *J. Fam. Pract.* **71**10.12788/jfp.0437 (2022). 10.12788/jfp.0437 35960943

[18] Murphy, K. R. et al. Development of the Asthma Impairment and Risk Questionnaire (AIRQ): a composite control measure. *J. Allergy Clin. Immunol. Pract.* **8**, 2263–2274.e5 (2020). 32387166 10.1016/j.jaip.2020.02.042

[19] Beuther, D. et al. Assessing the Asthma Impairment and Risk Questionnaire’s ability to predict exacerbations. In: *Monitoring Airway Disease*. European Respiratory Society PA3714 (2021).

[20] Chipps, B. E. et al. Assessing construct validity of the asthma impairment and risk questionnaire using a 3-month exacerbation recall. *Ann. Allergy Asthma Immunol.* **S1081-1206**, 00081–00083 (2022). 10.1016/j.anai.2022.01.035 35123077

[21] Yawn, B. Introduction of Asthma APGAR tools improve asthma management in primary care practices. *JAA*. Published online:1. August (2008). 10.2147/jaa.s3595 PMC3121335 21436980

[22] Yawn, B. P. et al. Use of Asthma APGAR tools in primary care practices: a cluster-randomized controlled trial. *Ann. Fam. Med.* **16**, 100–110 (2018). 29531100 10.1370/afm.2179 PMC5847347

[23] Nathan, R. A. et al. Development of the asthma control test: a survey for assessing asthma control. *J. Allergy Clin. Immunol.* **113**, 59–65 (2004). 14713908 10.1016/j.jaci.2003.09.008

[24] Schatz, M. et al. The minimally important difference of the Asthma Control Test. *J. Allergy Clin. Immunol.* **124**, 719–723.e1 (2009). 19767070 10.1016/j.jaci.2009.06.053

[25] Juniper, E. F., O’Byrne, P. M., Guyatt, G. H., Ferrie, P. J. & King, D. R. Development and validation of a questionnaire to measure asthma control. *Eur. Respir. J.* **14**, 902–907 (1999). 10573240 10.1034/j.1399-3003.1999.14d29.x

[26] Azevedo, P. et al. Control of Allergic Rhinitis and Asthma Test (CARAT): dissemination and applications in primary care. *Prim. Care Respir. J.* **22**, 112–116 (2013). 23412110 10.4104/pcrj.2013.00012 PMC6442752

[27] Ramsey, R. R. et al. A systematic evaluation of asthma management apps examining behavior change techniques. *J. Allergy Clin. Immunol. Pract.* **7**, 2583–2591 (2019). 30954644 10.1016/j.jaip.2019.03.041 PMC6776707

[28] Zhang, O., Minku, L. L. & Gonem, S. Detecting asthma exacerbations using daily home monitoring and machine learning. *J. Asthma* **58**, 1518–1527 (2021). 32718193 10.1080/02770903.2020.1802746

[29] Dabbs, W., Bradley, M. H. & Chamberlin, S. M. Acute asthma exacerbations: management strategies. *Am. Fam. Phys.* **109**, 43–50 (2024). 38227870

[30] Plaza Moral, V. et al. GEMA 5.3. Spanish guideline on the management of asthma. *Open Respir. Arch.* **5**, 100277 (2023). 37886027 10.1016/j.opresp.2023.100277 PMC10598226

[31] Chan-Yeung, M., Chang, J. H., Manfreda, J., Ferguson, A. & Becker, A. Changes in peak flow, symptom score, and the use of medications during acute exacerbations of asthma. *Am. J. Respir. Crit. Care Med.* **154**, 889–893 (1996). 8887581 10.1164/ajrccm.154.4.8887581

[32] Agusti, A. et al. Treatable traits: toward precision medicine of chronic airway diseases. *Eur. Respir. J.* **47**, 410–419 (2016). 26828055 10.1183/13993003.01359-2015

[33] Aldridge, R. E. et al. Effects of terbutaline and budesonide on sputum cells and bronchial hyperresponsiveness in asthma. *Am. J. Respir. Crit. Care Med.* **161**, 1459–1464 (2000). 10806139 10.1164/ajrccm.161.5.9906052

[34] Tattersfield, A. E. et al. Exacerbations of asthma: a descriptive study of 425 severe exacerbations. The FACET International Study Group. *Am. J. Respir. Crit. Care Med* **160**, 594–599 (1999). 10430734 10.1164/ajrccm.160.2.9811100

[35] Ghebre, M. A. et al. Severe exacerbations in moderate-to-severe asthmatics are associated with increased pro-inflammatory and type 1 mediators in sputum and serum. *BMC Pulm. Med.* **19**, 144 (2019). 31395050 10.1186/s12890-019-0906-7 PMC6688375

[36] Shrestha Palikhe, N. et al. Th2 cell markers in peripheral blood increase during an acute asthma exacerbation. *Allergy* **76**, 281–290 (2021). 32750154 10.1111/all.14543

[37] Miller, M. K., Lee, J. H., Miller, D. P. & Wenzel, S. E. TENOR Study Group. Recent asthma exacerbations: a key predictor of future exacerbations. *Respir. Med* **101**, 481–489 (2007). 16914299 10.1016/j.rmed.2006.07.005

[38] AsthmaStats: Uncontrolled Asthma among Adults, 2019 | CDC. August 12, 2022. Accessed March 31. https://www.cdc.gov/asthma/asthma_stats/uncontrolled-asthma-adults-2019.htm (2023).

[39] AsthmaStats: Uncontrolled Asthma Among Children With Current Asthma, 2018–2020 | CDC. August 22, 2022. Accessed March 31. https://www.cdc.gov/asthma/asthma_stats/uncontrolled-asthma-children-2018-2020.htm (2023).

[40] Bleecker, E. R., Gandhi, H., Gilbert, I., Murphy, K. R. & Chupp, G. L. Mapping geographic variability of severe uncontrolled asthma in the United States: Management implications. *Ann. Allergy Asthma Immunol.* **128**, 78–88 (2022). 34628005 10.1016/j.anai.2021.09.025

[41] Ding, B. & Small, M. Disease burden of mild asthma: findings from a cross-sectional real-world survey. *Adv. Ther.* **34**, 1109–1127 (2017). 28391549 10.1007/s12325-017-0520-0 PMC5427102

[42] Royal College of Physicians. Why asthma still kills; The National Review of Asthma Deaths (NRAD). RCP London. August 11, 2015. Accessed April 22. https://www.rcplondon.ac.uk/projects/outputs/why-asthma-still-kills (2024).

[43] Engelkes, M., Janssens, H. M., de Jongste, J. C., Sturkenboom, M. C. J. M. & Verhamme, K. M. C. Medication adherence and the risk of severe asthma exacerbations: a systematic review. *Eur. Respir. J.* **45**, 396–407 (2015). 25323234 10.1183/09031936.00075614

[44] Vähätalo, I. et al. 12-year adherence to inhaled corticosteroids in adult-onset asthma. *ERJ Open Res.* **6**, 00324–02019 (2020). 32211439 10.1183/23120541.00324-2019 PMC7086072

[45] Vähätalo, I. et al. Long-term adherence to inhaled corticosteroids and asthma control in adult-onset asthma. *ERJ Open Res.* **7**, 00715–02020 (2021). 33585657 10.1183/23120541.00715-2020 PMC7869602

[46] Paracha, R. et al. Asthma medication adherence and exacerbations and lung function in children managed in Leicester primary care. *npj Prim. Care Respir. Med* **33**, 12 (2023). 36966170 10.1038/s41533-022-00323-6 PMC10039953

[47] Papi, A. et al. Albuterol–Budesonide fixed-dose combination rescue inhaler for asthma. *N. Engl. J. Med.* **386**, 2071–2083 (2022). 35569035 10.1056/NEJMoa2203163

[48] Chipps, B. et al. Albuterol-budesonide Fixed-dose Combination (FDC) inhaler as-needed reduces progression from symptomatic deterioration to severe exacerbation in patients with moderate-to-severe asthma: analysis from MANDALA. *J. Allergy Clin. Immunol.* **151**, AB16 (2023).

[49] Israel, E. et al. Reliever-triggered inhaled glucocorticoid in black and Latinx adults with asthma. *N. Engl. J. Med*. Published online February 26. 10.1056/NEJMoa2118813 (2022). 10.1056/NEJMoa2118813 PMC10367430 35213105

[50] Beasley, R. et al. Evaluation of Budesonide-Formoterol for maintenance and reliever therapy among patients with poorly controlled asthma: a systematic review and meta-analysis. *JAMA Netw. Open* **5**, e220615 (2022). 35230437 10.1001/jamanetworkopen.2022.0615 PMC8889464

[51] Janjua, S. et al. Interventions to improve adherence to pharmacological therapy for chronic obstructive pulmonary disease (COPD). *Cochrane Database Syst. Rev.* **9**, CD013381 (2021). 34496032 10.1002/14651858.CD013381.pub2 PMC8425588

[52] Rootmensen, G. N., Van Keimpema, A. R. J., Jansen, H. M. & De Haan, R. J. Predictors of incorrect inhalation technique in patients with asthma or COPD: a study using a validated videotaped scoring method. *J. Aerosol. Med. Pulm. Drug Deliv.* **23**, 323–328 (2010). 20804428 10.1089/jamp.2009.0785

[53] Denholm, R., van der Werf, E. T. & Hay, A. D. Use of antibiotics and asthma medication for acute lower respiratory tract infections in people with and without asthma: retrospective cohort study. *Respir. Res.* **21**, 4 (2020). 31906966 10.1186/s12931-019-1233-5 PMC6945474

[54] Crompton, G. K. et al. The need to improve inhalation technique in Europe: a report from the aerosol drug management improvement team. *Respir. Med.* **100**, 1479–1494 (2006). 16495040 10.1016/j.rmed.2006.01.008

[55] Waljee, A. K. et al. Short term use of oral corticosteroids and related harms among adults in the United States: population based cohort study. *BMJ* Published online April 12:j1415. 10.1136/bmj.j1415 (2017). 10.1136/bmj.j1415 PMC6284230 28404617

[56] Price, D. B. et al. Adverse outcomes from initiation of systemic corticosteroids for asthma: long-term observational study. *J. Asthma Allergy* **11**, 193–204 (2018). 30214247 10.2147/JAA.S176026 PMC6121746

[57] Heatley, H. et al. Observational UK cohort study to describe intermittent oral corticosteroid prescribing patterns and their association with adverse outcomes in asthma. *Thorax* Published online December 27:thorax-2022-219642. 10.1136/thorax-2022-219642 (2022). 10.1136/thorax-2022-219642 PMC10447390 36575040

[58] Hew, M. et al. Cumulative dispensing of high oral corticosteroid doses for treating asthma in Australia. *Med. J. Aust.* **213**, 316–320 (2020). 32906192 10.5694/mja2.50758 PMC7589219

[59] Bleecker, E. R. et al. Systemic corticosteroids in asthma: a call to action from World Allergy Organization and Respiratory Effectiveness Group. *World Allergy Organ J.* **15**, 100726 (2022). 36582404 10.1016/j.waojou.2022.100726 PMC9761384

[60] O’Byrne, P. M. et al. Inhaled combined budesonide-formoterol as needed in mild asthma. *N. Engl. J. Med* **378**, 1865–1876 (2018). 29768149 10.1056/NEJMoa1715274

[61] O’Byrne, P. M. et al. Effect of a single day of increased as-needed budesonide–formoterol use on short-term risk of severe exacerbations in patients with mild asthma: a post-hoc analysis of the SYGMA 1 study. *Lancet Respir. Med.* **9**, 149–158 (2021). 33010810 10.1016/S2213-2600(20)30416-1

[62] Bateman, E. D. et al. As-Needed Budesonide–Formoterol versus Maintenance Budesonide in Mild Asthma. *N. Engl. J. Med.* **378**, 1877–1887 (2018). 29768147 10.1056/NEJMoa1715275

[63] O’Byrne, P. M. et al. Budesonide/formoterol combination therapy as both maintenance and reliever medication in asthma. *Am. J. Respir. Crit. Care Med.* **171**, 129–136 (2005). 15502112 10.1164/rccm.200407-884OC

[64] Scicchitano, R. et al. Efficacy and safety of budesonide/formoterol single inhaler therapy versus a higher dose of budesonide in moderate to severe asthma. *Curr. Med. Res. Opin.* **20**, 1403–1418 (2004). 15383189 10.1185/030079904X2051

[65] Rabe, K. F. et al. Budesonide/formoterol in a single inhaler for maintenance and relief in mild-to-moderate asthma: a randomized, double-blind trial. *Chest* **129**, 246–256 (2006). 16478838 10.1378/chest.129.2.246

[66] Calhoun, W. J. et al. Comparison of physician-, biomarker-, and symptom-based strategies for adjustment of inhaled corticosteroid therapy in adults with asthma: the BASALT randomized controlled trial. *JAMA* **308**, 987–997 (2012). 22968888 10.1001/2012.jama.10893 PMC3697088

[67] Panettieri, R. A. et al. Non-genomic effects of glucocorticoids: an updated view. *Trends Pharm. Sci.* **40**, 38–49 (2019). 30497693 10.1016/j.tips.2018.11.002 PMC7106476

[68] Alangari, A. A. Genomic and non-genomic actions of glucocorticoids in asthma. *Ann. Thorac. Med* **5**, 133–139 (2010). 20835306 10.4103/1817-1737.65040 PMC2930650

[69] Dubaybo, B. A. The care of asthma patients in communities with limited resources. *Res Rep. Trop. Med* **12**, 33–38 (2021). 33727880 10.2147/RRTM.S247716 PMC7954422

[70] Nanda, A. et al. Ensuring equitable access to guideline-based asthma care across the lifespan: Tips and future directions to the successful implementation of the new NAEPP 2020 guidelines, a Work Group Report of the AAAAI Asthma, Cough, Diagnosis, and Treatment Committee. *J. Allergy Clin. Immunol.* **151**, 869–880 (2023). 36720288 10.1016/j.jaci.2023.01.017

[71] Persaud, Y. K. Using telemedicine to care for the asthma patient. *Curr. Allergy Asthma Rep.* **22**, 43–52 (2022). 35107807 10.1007/s11882-022-01030-5 PMC8807679

[72] Guevara, J. P., Wolf, F. M., Grum, C. M. & Clark, N. M. Effects of educational interventions for self management of asthma in children and adolescents: systematic review and meta-analysis. *BMJ* **326**, 1308–1309 (2003). 12805167 10.1136/bmj.326.7402.1308 PMC161636

[73] Gibson, P. G. et al. Self-management education and regular practitioner review for adults with asthma. *Cochrane Database Syst. Rev*. CD001117. 10.1002/14651858.CD001117 (2023). 10.1002/14651858.CD001117 12535399

[74] Wilson, S. R. et al. Shared treatment decision making improves adherence and outcomes in poorly controlled asthma. *Am. J. Respir. Crit. Care Med.* **181**, 566–577 (2010). 20019345 10.1164/rccm.200906-0907OC PMC2841026

[75] Lugogo, N. et al. Real-world patterns and implications of short-acting β2-agonist use in patients with asthma in the United States. *Ann. Allergy, Asthma Immunol.* **126**, 681–689.e1 (2021). 33515710 10.1016/j.anai.2021.01.024

[76] Partridge, M. R., van der Molen, T., Myrseth, S. E. & Busse, W. W. Attitudes and actions of asthma patients on regular maintenance therapy: the INSPIRE study. *BMC Pulm. Med.* **6**, 13 (2006). 16772035 10.1186/1471-2466-6-13 PMC1483837

[77] McDonald, V. M., Harrington, J., Clark, V. L. & Gibson, P. G. Multidisciplinary care in chronic airway diseases: the Newcastle model. *ERJ Open Res.* **8**, 00215–02022 (2022). 35983538 10.1183/23120541.00215-2022 PMC9379354

