# Author Correction: Best practice advice for asthma exacerbation prevention and management in primary care: an international expert consensus

**DOI:** 10.1038/s41533-024-00411-9

**Published:** 2025-01-09

**Authors:** Neil Skolnik, Barbara P. Yawn, Jaime Correia de Sousa, María Mar Martínez Vázquez, Amanda Barnard, Wendy L. Wright, Austin Ulrich, Tonya Winders, Stephen Brunton

**Affiliations:** 1 Thomas Jefferson University, Philadelphia, PA USA; 2 Jefferson Health, Philadelphia, PA USA; 3 University of Minnesota, Minneapolis, MN USA; 4 University of Minho, Braga, Portugal; 5 University of the Basque Country, Leioa, Spain; 6 International Primary Care Respiratory Group (IPCRG), Scotland, UK; 7 Australian National University, Canberra, ACT Australia; 8 Wright & Associates Family Healthcare, Amherst, MA USA; 9 Partners in Healthcare Education, PLLC, Amherst, MA USA; 10 Primary Care Education Consortium, Winnsboro, SC USA; 11 Global Allergy & Airways Patient Platform, Vienna, Austria; 12 US Primary Care Respiratory Group, Winnsboro, SC USA

**Keywords:** Asthma, Therapeutics

Correction to: *npj Primary Care Respiratory Medicine* 10.1038/s41533-024-00399-2, published online 17 November 2024

“In the paragraph beginning ‘Use of ICS along with a SABA...’ in this article, the text ‘Of note, albuterol-budesonide is approved for mild asthma in the United States’ has been replaced with ‘Of note, albuterol-budesonide is approved for the as-needed treatment or prevention of bronchoconstriction and to reduce the risk of exacerbations in patients with asthma 18 years of age and older in the United States’. The original article has been updated.”

