# Supplementary material for: Best practice advice for asthma exacerbation prevention and management in primary care: an international expert consensus
Source: NPJ Prim Care Respir Med. 2024 Nov 17;34:39. doi: 10.1038/s41533-024-00399-2 (PMC11570618; doi:10.1038/s41533-024-00399-2)
Supplement: Supplementary file 1 — Supplementary Tables [file 41533_2024_399_MOESM1_ESM.docx]

Supplementary Tables

Asthma International Expert Consensus Panel Best Practice Advice Voting, Round 1

| **Voting Item** | **Responses** |
| --- | --- |
| “I agree with Best Practice Advice 1” | Yes: 56%, No: 0%, DNR: 44% |
| “I agree with Best Practice Advice 2” | Yes: 56%, No: 0%, DNR: 44% |
| “I agree with Best Practice Advice 3” | Yes: 56%, No: 0%, DNR: 44% |
| “I agree with Best Practice Advice 4” | Yes: 56%, No: 0%, DNR: 44% |
| “I agree with Best Practice Advice 5” | Yes: 56%, No: 0%, DNR: 44% |
| “I agree with Best Practice Advice 6” | Yes: 56%, No: 0%, DNR: 44% |
| “I agree with Best Practice Advice 7” | Yes: 56%, No: 0%, DNR: 44% |
| “I agree with Best Practice Advice 8” | Yes: 56%, No: 0%, DNR: 44% |
| “I agree with Best Practice Advice 9” | Yes: 56%, No: 0%, DNR: 44% |
| “I agree with Best Practice Advice 10” | Yes: 56%, No: 0%, DNR: 44% |

DNR, did not respond

Panel composed of 9 members

Asthma International Expert Consensus Panel Best Practice Advice Voting, Round 2

| **Voting Item** | **Responses** |
| --- | --- |
| “I agree with Best Practice Advice 1” | Yes: 100%, No: 0% |
| “I agree with Best Practice Advice 2” | Yes: 89%, No: 11% |
| “I agree with Best Practice Advice 3” | Yes: 100%, No: 0% |
| “I agree with Best Practice Advice 4” | Yes: 100%, No: 0% |
| “I agree with Best Practice Advice 5” | Yes: 100%, No: 0% |
| “I agree with Best Practice Advice 6” | Yes: 100%, No: 0% |
| “I agree with Best Practice Advice 7” | Yes: 100%, No: 0% |
| “I agree with Best Practice Advice 8” | Yes: 100%, No: 0% |
| “I agree with Best Practice Advice 9” | Yes: 100%, No: 0% |
| “I agree with Best Practice Advice 10” | Yes: 100%, No: 0% |

Panel composed of 9 members
